# The British Journals: Toxicology

**Published:** 1839-10

**Authors:** 


### TOXICOLOGY.

*Experiments with the Wourali Poison. With Remarks by* DR. CLANNY.

THIS poison is chiefly manufactured by the Accaaway Indians, who inhabit British Guiana, between the rapids of the Demerara river and the high mountains of the interior. The materials of which the poison is composed are unknown, and it is not unlikely but the secret will die with its possessors. So many reports had been circulated relative to the nature and properties of this poison, that in 1813, Mr. Waterton, a gentleman of independent property, who had visited Demerara purely through love of science, undertook, at his own expense, to travel 800 miles into the interior, for the purpose of ascertaining the precise facts. The privations and toil he underwent are detailed in his "Wanderings in South America," a work replete with interesting narratives and not a little humour. He obtained a considerable quantity of the poison, and on his return to England, experiments were tried on various animals: among others was a female ass, about three years old, upon whom the experiment we shall subsequently record was practised, and the creature recovered so as to be able to stand up in four hours. The creature was, at the request of the Duke of Northumberland, sent to Walton Hall (Mr. Waterton's residence), but it was some time before the constitution recovered the shock, though she ultimately became strong and healthy, and lived to the age of twenty-seven years, dying on the 15th of February last, through sheer decay of nature. Thus the point being fully proved that restoration to life could be effected after the animal functions had been entirely suspended by the deadly nature of the poison, it was conjectured that the wourali might be applied in certain cases of disease, so that first with the total suspension of animation, and next the succeeding restoration, a new existence might be imparted.

"Viewing the disease in horses as the result of irritation of the nervous system, Mr. Sewell conjectured that if a horse in tetanus were destroyed by poison, which acts by suppressing nervous power, and life were then to be restored by artificial respiration, the nervous system, on reanimation taking place, might possibly be free of the original morbid irritation. Reasoning thus, Mr. Sewell tried the following singular practice: A horse, suffering from a severe attack of the tetanus and locked jaw, the mouth being too firmly closed to admit the introduction of either food or medicine, was inoculated on the fleshy part of the shoulder with an arrow point coated with the wourali poison; in ten minutes apparent death was produced. Artificial respiration was immediately commenced and kept up about four hours, when reanimation took place, the animal rose up apparently perfectly recovered, and eagerly partook of hay and corn. He unluckily was too abundantly supplied with food during the night. The consequence was over distention of the stomach, of which the animal died the following day, without, however, having the slightest recurrence of tetanic symptoms."*

Other experiments have, we believe, been tried on animals at various times, with similar results, but no attempt that we know of has as yet been made on the human frame. In what way the poison acts has not been accurately discovered. A recent case of Hydrophobia at Nottingham induced a desire to ascertain whether it might not be successfully applied in hydrophobia. A request was sent to Mr. Waterton, at Walton Hall, to aid with his usual kind and generous feeling; that gentleman promptly attended, but unhappily poor Phelps expired before his arrival. Ever willing, however, to promote the advancement of science, Mr. Waterton engaged to exhibit the experiment upon animals, before the surgical and medical profession; and on Monday last, being the day appointed, the schoolroom, at the entrance to the Chapel Yard, in St. James's street, was selected for the occasion.

Exp. i. A brindled dog of good size was brought in for the purpose of testing the effects of the poison. An incision was made in the animal's left side, and an arrow-head, covered with the wourali, introduced; but, for a quarter of an hour, it manifested no symptoms of distress; at the end of that time, however, the action of the heart became irregular and tumultuous, and the pulse rose to 130, irregular and intermitting. At the expiration of 34 minutes, the dog showed indications of distress by twitchings and feebleness of the legs. At 36 minutes, the poison was operating powerfully; the convulsions were constant; the pupils of the eye greatly dilated; and the animal, unable to stand, fell down. The arrow-head was withdrawn. At 40 minutes, the struggling increased, and the pulse was very irregular, the respiration getting more difficult. At 42 minutes, the bladder was acted upon. At the end of 52 minutes, respiration had entirely ceased: there was a feeble action of the heart, the limbs supple. Five minutes more were allowed to elapse, and the body was opened. The heart was full of fluid blood,

* Mayo's Outlines of Physiology.

and, on being pricked with the point of a scalpel, gave not the slightest indication of irritability.

Exp. ii. A female ass, eight years old, was next tried. The animal was in rather poor condition, but, previous to operating, the action of the heart was regular, the pulse at 62.

At 9h. 6m. an incision was made in the neck of the ass, near the right shoulder, and an arrow-head, strongly poisoned, introduced into the wound. At 9h. 12m. the pulse was at 60, and continued uninterruptedly the same for five or six minutes longer, when it began to increase. At 9h. 20m. the pulse was irregular and had risen to 72, the respirations at 14 in a minute: at this time the pupils of the eye were becoming dilated. At 9h. 32m. the pulse was still at 72, the animal manifesting symptoms of uneasiness. At 9h. 36m. the pulse had risen to 84, and the creature repeatedly lifted its fore legs, which trembled, and the next minute it fell down, being exactly 31 minutes from the time of introducing the poison. There was a slight struggling, but the muscular power soon ceased. The pulsation was imperceptible, except a slight fluttering at the heart, and animation appeared to be extinct. The animal was then raised upon a table. An opening was immediately made in the trachea, and at 9h. 41m. artificial respiration was commenced.

The apparatus for producing respiration is a double pair of bellows, keeping up a constant current of air. To this a leathern pipe was made to fix over the nozzle; the other end of this pipe is attached to a tube that is introduced, by a bend, into the trachea, but easily removed in a moment, and a circular pad upon the tube, near the bend, is drawn pretty tight over the wound, so as to prevent an escape of air. Five operators are required: one to keep the bellows going, another to shift the part of the pipe on the tube, two to the body of the animal, and a fifth to the nostrils. The wind is forced by the bellows through the leather pipe and tin tube into the lungs; the moveable part of the leather pipe is then withdrawn from the tin tube, and the two individuals, appointed for the purpose, press upon the animal, so as to force the wind out again through the tube, the nostrils being stopped. The repetition of this process must be equal in time to the usual respirations of human beings in a state of health, or from sixteen to eighteen in a minute, so that some idea may be formed of the labour required.

At 9h. 44m. the pulsations became once more distinct, as high as 72, and continued varying, for seven hours, from 56 to sometimes 100, but most frequently at between 70 and 80. No other symptoms of life were at all displayed. Natural respiration was wholly suspended during the entire time, but about four o'clock there was evidently a slight muscular motion. Enemas of warm water and turpentine had been administered, and the bowels were freely acted upon. Dr. Davidson had also inserted on the right shoulder about 4 grains of strychnine: and turpentine had been used, as well as ammonia applied to the nostrils, but the room was so crowded by visitors that it was next to impossible accurately to take notes. About this time (four o'clock) the creature displayed symptoms, by the agitation of the nostrils, of returning natural respiration. At 4h. 38m. its natural respiration was free, and the artificial respiration was discontinued. Soon after the animal was laid on some straw before a large fire, where it continued in a very doubtful condition through the night. Every attention was paid to it. At 8h. (in the evening) it drank some water, and horns of gruel were occasionally given. It sometimes struggled a little with its legs, as if desirous of rising, but could not well raise its head. The pulse was feeble, and varying from 86 to 104. Towards the morning it breathed with great labour and difficulty, and apprehensions were entertained that it must sink; but between five and six o'clock the tube was removed from the trachea, and some mucus and blood was voided at the incision. Some lint was applied to the wound, and at seven o'clock it was sewed up.

At eight o'clock it was raised on its belly and seemed pleased with the change of position. A warm bran mash was offered in a bucket, of which it ate with much ease. Its breathing became more free, and about nine o'clock it was raised on its legs, but they were unable to sustain it, particularly the near foreleg, which was contracted and paralyzed. It was taken out into the yard for a couple of hours, but the cold was too intense, and it was again brought before the fire. At noon its pulsation was at 80, and it ate freely of mash and hay, and repeated horns of gruel were given to it. The creature continued improving through the day, but retarded the progress of recovery by ineffectual struggles to rise.

Exp. iii. During the afternoon of Monday, a second dog was tried and expired in about half an hour. It was intended to try artificial respiration, and the trachea was opened, but the bellows were inadequate to the operation, and the attempt was abandoned.

Exp. iv. At eight o'clock (Tuesday), a male ass, about five years of age, was brought into the room; it appeared very vigorous, but with ventral hernia on the right side. It stood perfectly quiet, and the action of the heart was at 36. Mr. Attenburrow, the surgeon, cut into the trachea at 8h. 4m., and an incision was made on the fore part of the right shoulder, into which, at 8h. 6m. Mr. Waterton inserted an arrow-head with the wourali on it, the quantity being not quite one fourth of that used upon the ass yesterday. The creature struggled a little whilst the operation was going on, but as soon as it was over remained perfectly quiet.

At 8h. 16m. the pulse was at 66; and about four minutes afterward, the pupil of the eye become gradually dilated, though the animal stood perfectly patient, manifesting no symptoms of uneasiness.

At 8h. 30m. the pulse was at 62, and at that moment the ass operated on yesterday commenced with vigour to eat a mash; and some oats being offered to the one operating upon, it ate them very readily and continued to do so.

At 8h. 38m. spasmodic twitchings were evident in the abdomen, the animal still eating and apparently not much distressed. The next minute the legs trembled, and at 8h. 40m. the creature fell on its belly, but tried to rise again.

In two minutes more the twitching increased, with occasional struggles, but not very violent, and the animal lay down upon its left side, where it was suffered to remain, a bed of straw having been prepared for the purpose in the middle of the room. The tin tube was introduced into the trachea; the insertion caused the animal to kick a little, but it still continued chewing the oats.

At 8h. 44m. the action of the heart was regular, but the respiration convulsive and difficult, with repeated gaspings. It struggled occasionally, and at 8h. 49m. the pulse was 54, with spasm and difficult respiration, but the pupil of the eye not so much dilated.

At 8h. 54m. the arrow-head was withdrawn from the incision, and no poison remained upon it. The pulse continued the same, and the respirations were 44 in a minute; the vision pretty perfect.

At 8h. 57m. there was a gurgling in the throat and the respiration less frequent. Two minutes afterwards the animal struggled hard, kicked out with its hind legs, and at nine o'clock there was a tremor of the muscles, the respiration extremely difficult; the vision remained tolerably good.

At 9h. 4m. the action of the heart was strong but irregular, and the next minute the labour for breath was extremely difficult, the gaspings frequent, accompanied by spasm.

At 9h. 7m. respiration ceased, the vision entirely gone, the action of the heart was energetic, the extremities warm, throbbing of the carotid artery strong. The apparatus was put in motion, and artificial respiration commenced at an average of 18 a minute.

At 9h. 20m. the action of the heart was very feeble, and in other parts the pulsation was imperceptible. The artificial respiration was kept up on an average of 16 or 18; there was a spasmodic twitching of the eyebrows, and an occasional slight muscular motion of the tongue.

At 9h. 30m. the extremities began to get cold, but there was no rigidity in the limbs.

At 9h. 45m. the heart's action was quick but very feeble.

At 9h. 55m. the extremities were colder, but a warmth was still preserved in the region of the heart, and behind the ear, near the root.

At 10h. 1m. the animal made spontaneous efforts to breathe; the heart's action was energetic but not powerful, but no other pulsation perceptible.

At 10h. 10m. the lips were slightly agitated, the motion of the tongue was more active, and temperature of body improved.

At 10h. 25m. the nostrils gave indications of restored respiration, and the artificial respiration was kept up at 16 per minute. No pulsation perceptible except in the action of the heart.

At 10h. 27m. natural respiration recommenced.

At 10h. 36m. the ears were slightly agitated, and symptoms of returning vision.

At 10h. 40m. the respiration much hurried and irregular.

At 10h. 41m. the artificial respiration was discontinued.

At 10h. 44m. the natural respirations were 38.

At 10h. 50m. the natural respirations were 56, the breath warm, slight bleeding from the lower nostril, pulse very irregular, spasm on hind legs.

At 11 o'clock the natural respirations were 58.

At 11h. 5m. pulsations of the heart quick and laborious. The door of the room was closed with violence, and the noise produced a startling and quick motion of the whole body.

At 11h. 10m. the respirations 50, the ears cold.

At 11h. 17m. the pulsation at the heart was 60 and very feeble, barely perceptible in the arteries, and not possible to count.

At 11h. 22m. the pulsation of the heart was at 80, respiration improving, and muscular power increasing.

At 11h. 25m. some water was dropped upon his mouth, which he licked in with his tongue and swallowed, but the effort produced struggles.

At 11h. 30m. the pulse was at 52.

At 11h. 32m. the animal struck out with his legs rather violently.

At 11h. 25m. the respiration was 38.

At 11h. 40m. the respiration was 56; the mouth was moistened with water, producing strong muscular agitation.

At 11h. 55m. the pulse at 65 and feeble.

At 12 o'clock the respiration became more feeble, the animal heat was diminished, and the pulsation at 60; the eye not so susceptible of light.

At 12h. 5m. some gruel was poured upon the mouth, which produced violent muscular action; some hay was offered, which it smelt and attempted to eat.

At 12h. 13m. more hay was presented, which, in his attempts to eat, caused a spasmodic action of the respiratory organs.

At 12h. 25m. the pulse was at 65.

At 12h. 36m. the pulse was at 55, feeble; and the animal, attempting to cough, forced a quantity of mucus through the tin tube which remained in the trachea. It was evident that, by continued coughing, something was irritating the animal's throat, below where the incision in the trachea was made; and at 12h. 50m. a considerable quantity of mucus, tinged with blood, was forced through the tube; this continued at intervals for some time, the ass ejecting the mucus with difficulty.

At 1h. 40m. the pulsation was 60; and a piece of straw having got into the tube irritated the animal, who, in trying to get rid of it, struck the tube against the ground; the suddenness of the pain caused the creature to rise up upon his legs, and in a few minutes he walked about with a tolerable degree of strength. The tube was removed from the trachea, and some water being given it in a bucket it drank freely; the mucus continued to annoy it for some time longer, but at last it discharged the principal portion, breathed more readily, and manifested much greater ease.

At 2h. 35m. the pulse was 64, and the bowels and bladder acted upon pretty freely.

At 4 o'clock it began to eat some warm mash, and afterwards some hay; the wound in the trachea closed, so that no air was admitted, and the animal went on gradually gaining strength and becoming lively.

At five o'clock a person mounted his back, and he bore him without the least difficulty across the room. Since then the ass manifests every symptom of liveliness, strength, and even playfulness, betraying no signs of having been operated on.

Our reporter visited the patients yesterday (Thursday) afternoon, and found the one last operated upon trotting about the room as lively as possible. Indeed, the attendant informed him that, but for the coldness of the weather, he would have been turned out to grass that morning. The other (the medical gentlemen who examined them in the early part of the day said) was considerably better, though not yet able to stand for a long time at once, owing to the paralysis with which the near foreleg is affected; but the most sanguine hopes are entertained of its ultimate recovery.

The ass first operated upon died on Sunday morning last, about six o'clock. A *post-mortem* examination was consequently appointed to take place on Monday afternoon, at two o'clock. The operation was performed at the Medical School, by Mr. Sibson, of the General Hospital, in the presence of several of the faculty. On opening the animal, four or five ounces of reddish fluid were found in the pericardium. There were also four or five ounces in the cavity of the pleura. The lungs were in a firm state. The internal lining of the heart was very red—the surface quite pale—very little blood in the interior—some gas in the blood-vessels on its surface. There was no positive inflammation going on in the lungs; the lung most engorged was that on the side on which it had lain, and in that there was no more engorgement than what was apparently the effect of its lying on that side. There were no signs of vascularity or inflammation in the peritoneum. In fact, inflammation was not observed in any part, if we except a little in the immediate neighbourhood of the wound made in the trachea, for the introduction of the pipe, and that apparently only sufficient to have closed the wound, had the animal lived, and the parts where the wourali and the strychnine had been introduced, and those only superficial. The brain was found perfectly healthy, as well as the spinal marrow. The bowels were very full of food and wind, but exhibited no external inflammatory signs whatever. In short, there were no morbid appearances in the body to account for its death. The animal died of complete debility. It never overcame the shock it received from being in the unnatural state in which it was for seven hours. It was the opinion of several of the medical gentlemen, that it had never been so well since it was led out into the air the day after the operation was performed. The hair had come off in several parts of the body, which was caused by some turpentine having been spilt upon the table, and having got through the hair upon the skin. These were all the appearances noticed.

*Nottingham Journal. April* 12*th and* 19*th*, 1839.

Remarks by Dr. Clanny. Since the publication of the well-conducted and valuable experiments upon decapitated and living animals, elucidatory of the phenomena of respiration, performed by Sir Benjamin Brodie and Mr. Broughton, I have not been so much interested upon perusal of any others as those performed at Nottingham with the Wourali poison.

Since my discovery of the gases circulating in our arterial and venal blood, we have been enabled to explain the phenomena of the action of the Wourali poison in such cases. We find that, in the first experiment, artificial respiration was not employed, and that the deleterious effects of the poison were allowed to take their course upon the sensorium commune; hence the absorption of atmospherical air into the extreme branches of the pulmonary veins was prevented.

In the second experiment, the animal's life was saved by artificial respiration, and thus a certain portion of atmospherical air was absorbed into the systemic system.

The third corroborates the previous experiment, and requires no comment.

From the above-named discovery, made in the year 1834, we can also readily understand the manner in which the fœtus in utero receives a suitable quantity of oxygen from the parent. *Lancet. May* 18, 1839.

